# Judicial Department

**Published:** 1856-01-01

**Authors:** 


### Part Fourth.

### JUDICIAL DEPARTMENT.

### AN EXPOSITION OF THE LAW RELATING TO CHANCERY LUNATICS.

LUNATICS, for the purposes of legislation, have been divided into three classes:—

1. Persons found lunatic by inquisition, often called Chancery lunatics.
2. Persons not so found lunatic, but who are placed under the restraint of a lunatic asylum ; *i.e.*, in private asylums, or as single patients, under certificates.

3. Pauper lunatics.

The various statutes relating to the first class were consolidated and amended by "The Lunacy Regulation Act, 1853" (16 and 17 Vict. cap. 70) ; "An Act for the Regulation of Proceedings under Commissions of Lunacy, and the Consolidation and Amendment of the Acts respecting Lunatics so found by Inquisition, and their Estates."

The statutes relating to the second class were consolidated and amended by the 8 and 9 Vict. c. 100, "An Act for the Regulation of the Care and Treatment of Lunatics," and which has since been amended by the "Lunatics' Care and Treatment Amendment Act, 1853" (16 and 17 Vict. c. 96), "An Act to Amend an Act passed in the ninth year of her Majesty, for the Regulation of the Care and Treatment of Lunatics."

The statutes regulating the third class were consolidated and amended by "The Lunatic Asylums Act, 1853" (16 and 17 Vict. c. 97), "An Act to Consolidate and Amend the Laws for the Provision and Regulation of Lunatic Asylums for Counties and Boroughs, and for the Maintenance and Care of Pauper Lunatics in *England*."

We have frequently in this journal considered the state of the law as to the second class, and in vol. vi. p. 590, we enumerated the various alterations and amendments made by the 16th and 17th Vict. c. 96, with reference to private asylums and single patients. We propose now to consider the state of the law relating to Chancery lunatics.

Previous to the year 1842, the practice in lunacy was conducted according to the then dilatory and expensive fashion of Chancery proceedings, in the offices of the Masters in Chancery; but in that year a measure was introduced by Lord Lyndhurst, and was passed,* which had the effect of greatly diminishing the delay and expense of proceedings in lunacy. The following were the principal alterations effected by that statute and the general orders framed under its authority. Two Masters in Lunacy were specially appointed to execute commissions of lunacy, and to conduct the business of the lunacy department of the court, which before devolved upon the Masters in Chancery. By this arrangement the time occupied in the execution of commissions, as well as the expense, was considerably reduced; the expenses of proceedings after the inquisition were also much reduced by the diminution of the number of petitions, orders, and reports, the Masters having jurisdiction to inquire and report in many cases without any order of reference; and the general orders in many cases provided for measures which previously would have required a special order in each case. These alterations having worked well, both simplifying the practice and diminishing expense, Lord Lyndhurst, in 1852, introduced into the House of Lords a bill for effecting further improvements, intituled "An Act to diminish the Expense of Proceedings under Commissions *de lunatico inquirendo*."

The expense of lunacy proceedings may be divided into two classes: 1. The expenses incurred in obtaining the decision of a competent

* 5 and 6 Vict. c. 84.

legal tribunal as to the lunacy; 2. The expenses connected with the care of the lunatic and the management of his estate after he has been declared lunatic. The first class comprehended the costs of suing out a commission of lunacy under the great seal; the fees of a numerous jury, usually more than twelve in number, and when special jurors paid a guinea each per diem; the expense of witnesses and fees of counsel when retained; and all other expenses incurred about the inquisition, as well as the traverse, if the inquisition were traversed, but which seldom occurred. The expense of the inquisition in a contested case must always be considerable, but much unnecessary expense arose from the form of the commission requiring the verdict to determine, not simply the fact of the lunacy, but also the date when the lunacy commenced. This latter inquiry might run through many years, requiring numerous witnesses at great expense, and its advantage is not apparent; for, if the lunatic has contracted a debt subsequently to the period to which the verdict applies, the creditor may nevertheless prosecute his claim for it, or, if he has made a will or executed any deed during the same period, any person interested may attempt to establish the sanity of the lunatic at the date of such will or deed. The finding of the verdict of lunacy is undoubtedly *primâ facie* evidence against the debt, or will, or any other transaction subsequent to the date fixed by the verdict, but it is not conclusive against any person who was not a party to it.*

With respect to the second class of expenses of lunacy proceedings, the appointment of committees by grant of the custody of the person and estate of the lunatic under the great seal formed one item; but the expenses under this head were occasioned chiefly by the multiplicity of petitions, orders, and reports required to give effect to the various matters of constant occurrence in the administration of a lunatic's estate. Lord Lyndhurst proposed to meet this by striking at the root of the evil, and suggested the adoption of a mode of proceeding somewhat similar to the principle of the Joint-Stock Companies' Winding-up Acts† and the Irish Chancery Act‡—viz., that the Master's report should be dispensed with, and an order should be made in the first instance, not by the Lord Chancellor, but by the Master in Lunacy, whose order should be binding and conclusive on all parties, if not appealed from to the Lord Chancellor within a given time.§ There can be little question that the simplification of the mode of procedure is the true means of obviating both delay and expense, for both are chiefly caused by the circuitous mode formerly, according to the practice, unavoidable in almost every proceeding. The following is a list of suggestions made by Lord Lyndhurst for simplifying the practice and diminishing the expense of proceedings in lunacy:—

1. That the fiat of the Lord Chancellor should be substituted for, and have the same effect as a commission of lunacy.||
2. That the jury should be dispensed with, and the Master in Lunacy

* 120 Hansard, 353. † 11 and 12 Vict. c. 45; and 12 and 13 Vict. c. 108.

‡ 13 and 14 Vict. c. 89. § 120 Hansard, 351.

|| Ibid. 351.

should by himself decide the question of lunacy, unless the alleged lunatic required the verdict of a jury.*

3. That the inquiry as to the lunacy should be confined to the period when the inquisition is made, or otherwise reduced within narrow limits, instead of being carried back to the commencement of the lunacy.†
4. That the Master in Lunacy should be invested with a discretionary power to limit the costs of opposition to the inquiry as he should think proper, with the view of checking such opposition by improper persons for the sake of obtaining the costs.‡
5. That the traverse should be altogether disallowed *as a matter of right*, and be at the discretion of the Lord Chancellor.§
6. That the grant of the custody of the person and estate of the lunatic under the great seal should be discontinued, and that the Lord Chancellor's order should have the same effect as a grant.‖
7. That the Master's report should be dispensed with, and an order should be made in the first instance by the Master, which should be binding and conclusive on all parties, if not appealed from to the Lord Chancellor within a given time.¶
8. That the powers given by the Act 8 and 9 Vict. c. 100, § 94—98 (under which, in certain cases, the Master in Lunacy, without a jury, decided as to the lunacy of a party confined as a lunatic,) should be extended so as to correspond with the powers given by a commission of lunacy.**

These were valuable suggestions, and, as we shall see hereafter, most of them have practically been carried into effect by the provisions of the 16 and 17 Vict. c. 70; indeed, the only room for debate as to their soundness would be on the 2nd and 5th suggestions, as to which the Briton's veneration for trial by jury and the right of appeal might raise a doubt in the mind of one not informed on the subject. In submitting his suggestions to the House of Lords, Lord Lyndhurst mentioned that "he had intended to propose them as soon as he had acquired experience of the working of his bill of 1842. He had, however, given up the custody of the great seal before he could carry his intentions into effect,"†† and concluded by stating that "he knew his noble and learned friend, the Lord Chancellor (St. Leonards), was so anxious to introduce, as far as possible, perfection in every department of the administration of justice under his control, that he might safely leave the case in his hands. He (Lord Lyndhurst) was desirous of consulting and co-operating with him, for the purpose of carrying into effect as many of the suggestions he had made as he thought could be adopted consistently with the security of the lunatic and the advantage of the public."‡‡ Lord St. Leonards intimated that when he had disposed of the measures of Chancery reform then under consideration, he should endeavour to meet Lord Lyndhurst's suggestions.§§ The bill introduced by Lord Lyndhurst was not advanced during that

* 120 Hansard, 355. † Ibid. 352. ‡ Ibid. 353. § Ibid. 357.

‖ Ibid. 352. ¶ Ibid. 351. ** Ibid. 358.

†† Ibid. 350. ‡‡ Ibid. 359. §§ Ibid. 361.

session, but in the following session Lord St. Leonards, in his speech from the woolsack, on the administration of justice,* announced that his proposed measures of reform would include the law of lunacy, and the three several acts before mentioned (16 and 17 Vict. cc. 70, 96, 97) were introduced and passed.

The "Lunacy Regulation Act, 1853" (16 and 17 Vict. c. 70,) repealed the various acts and portions of acts mentioned in the schedule, and re-enacted so much thereof as was to be retained. This act regulates proceedings under commissions of lunacy, and the management of lunatics so found by inquisition and their estates. The Masters in Lunacy act under a general commission, and instead of a special commission of lunacy being directed to them in each case, they proceed under an order of the Lord Chancellor or Lords Justices,† directing an inquiry into the lunacy; the jury is dispensed with, except where the alleged lunatic demands a jury. Committees are appointed by a simple order, instead of a grant, under the great seal; the practice requiring frequent petitions, reports, and orders, is much simplified; a check is placed upon unnecessary costs; the Masters have enlarged powers for preventing delay in proceedings; the Lord Chancellor's jurisdiction over the lunatic's property is extended so as to provide for almost any imaginary contingency; the fees are reduced to three, which are small in amount, and are collected by means of stamps; and a moderate percentage is paid on all incomes of 100*l.* and upwards, for defraying the official expenses incident to the administration of lunatics' estates, which includes the percentage heretofore collected under the 3 and 4 Wm. IV. c. 36, for meeting the expense of visiting lunatics. The act extends to England and Wales, and to Ireland where the same is specifically mentioned, § 4; and took effect from the 28th Oct. 1853, § 5.

We will now consider, more in detail and in their order, some of the principal alterations effected by this statute.

The act recites that it would greatly facilitate the simplification and improvement of the practice in lunacy, and would be attended with convenience, and with a saving of expense to the estates of lunatics, that the charges incident to the administration of the estates of lunatics under the authority of the Lord Chancellor, should be defrayed in part by means of a percentage, graduated in an equitable manner as between the richer and poorer estates, and in part by means of fees on proceedings; and by § 26 it is enacted, that a percentage on the clear annual incomes of all lunatics shall be paid according to the following rates:—

The rate of four per centum for each clear annual income amounting to 100*l.* and not amounting to 1000*l.*, but so that no larger sum be payable in any such case, in any one year, than 30*l.*;

The rate of three per centum for each clear annual income amounting to 1000*l.*, and not amounting to 5000*l.*, but so that no larger sum be payable in any such case, in any one year, than 100*l.*; and

The rate of two per centum for each clear annual income amounting

* 123 Hansard, 181.

† The Lords Justices of the Court of Appeal in Chancery, under warrant from the Crown, have concurrent jurisdiction with the Lord Chancellor in Lunacy.

to 5000*l.* or upwards, but so that no larger sum be payable in any such case, in any one year, than 200*l.*

And by § 29, all fees heretofore payable in relation to proceedings in lunacy are abolished, and in lieu thereof the following fees only are to be paid:—

For each order or fiat of the Lord Chancellor . . . . . . 2*l.*

For each report or certificate of the Masters and Taxing Masters . . . . . . . . . . . . . . . . . . 1*l.*

For attending any court by the Clerk, per diem . . . . . 1*l.*

And instead of the exorbitant charges for copies, &c., the charge for all engrossments, transcripts, and copies of documents and papers will be the actual amount paid to the stationer. But by § 30, the Lord Chancellor has power to vary the rates of percentage, so that they do not exceed the rates fixed by § 26; and he may also vary the fees. The percentage and fees are collected by means of stamps, under the provisions of the 15 and 16 Vict. c. 87, § 6—13 (§ 31); and by § 32 the Lord Chancellor may, if he think fit, exempt small properties not exceeding 700*l.* in respect of corpus, or 50*l.* per annum in respect of income, from payment of fees and percentage; though it may be observed, that § 26, which regulates the percentage, does not authorize the payment of any percentage on incomes less than 100*l.*

The principle of levying a percentage for defraying the expenses of administering the estates of lunatics is only just, and we are glad to find it at length adopted. The fees, now so few and of such small amount, even with the addition of the percentage, will no longer be a burden on small estates. If it is right that there should be any tax on the administration of justice, it is only reasonable that the amount levied should bear some relation to the value of the subject matter in suit or under administration; and here it cannot be said that the rich estate is unfairly made to bear the expense of administering the poor estate, for 200*l.* is the largest amount of percentage that can be paid in any one year for any estate, and that only on an income of 10,000*l.* or upwards.*

With respect to the commission and inquisition, very considerable alterations are made by § 38—54. In lieu of the commission heretofore issued specially in each case of alleged lunacy, a general commission has been issued under the great seal, directed to the Masters in Lunacy, who, under its authority, proceed in each case of alleged lunacy, concerning which the Lord Chancellor directs them to inquire,

* The late Lord Truro stated in the House of Lords, "that there are 497 lunatics' estates under the jurisdiction of the great seal. The aggregate income of those estates is £317,493. Of those

| | | | | | |
|---|---|---|---|---|---|
| 100 are under | £100 | a-year. | 27 are under | £1500 | a-year. |
| 112 ,, | 200 | ,, | 7 ,, | 2000 | ,, |
| 57 ,, | 300 | ,, | 14 ,, | 3000 | ,, |
| 46 ,, | 400 | ,, | 9 ,, | 4000 | ,, |
| 30 ,, | 500 | ,, | 4 ,, | 5000 | ,, |
| 31 ,, | 600 | ,, | 3 ,, | 6000 | ,, |
| 13 ,, | 700 | ,, | 2 ,, | 7000 | ,, |
| 16 ,, | 800 | ,, | 3 upwards of | 7000 | a-year." |
| 23 ,, | 1000 | ,, | | | |

120 Hansard, 367.

§ 39. Where the alleged lunatic is within the jurisdiction, he has notice of the presentation of the petition for inquiry, and he may, by notice signed by him, demand an inquiry before a jury, § 40;* upon which the Lord Chancellor, by his order for inquiry, directs the return of a jury, unless he be satisfied, by personal examination of the alleged lunatic, that he is not mentally competent to form and express a wish for an inquiry before a jury, for which purpose the Lord Chancellor may, where he deems it necessary, require the alleged lunatic to attend him, § 41; but where he does not demand an inquiry before a jury, or the Lord Chancellor is satisfied by personal examination of him that he is not mentally competent to form and express a wish for a jury, and it appears to the Lord Chancellor upon consideration of the evidence adduced before him on the petition for inquiry, and of the circumstances of the case, so far as they are before him, to be unnecessary or inexpedient that the inquiry should be before a jury, and he accordingly does not in his order for inquiry direct the return of a jury, then the Masters, by virtue of their general commission and under the order for inquiry, without a jury, personally examine the alleged lunatic, and take such evidence, upon oath or otherwise, and call for such information as they may think fit or the Lord Chancellor may direct, in order to ascertain whether or not the alleged lunatic is of unsound mind, and certify their finding thereon, § 42; and the Master's certificate as to the state of mind of the alleged lunatic is to be deemed an inquisition, and is to be of the same force and effect to all intents and purposes, and is to be returned, filed, and proceeded on in the same manner in all respects as an inquisition taken upon the oath of a jury, § 44.

This is an important change, and may be objected to by those who would allow no substitute for trial by jury; but its object is to meet cases of obvious insanity. Lord St. Leonards said he "agreed with Lord Lyndhurst that it would be undesirable to abolish altogether trial by jury in cases of disputed lunacy—to say that they would take away the liberty and estate of the subject without the benefit of a jury was quite impossible; but this he thought was perfectly clear, that there were a great many cases where there was no question as to the insanity, in which such a trial was an idle waste of money, at the expense of the lunatic himself whom you were seeking to protect. You protected him by this trial indeed, but it was at the expense of his property; and you left him without the means of maintenance by a measure which was not of the slightest benefit to him. This was especially true as regarded lunatics possessed of small property."† But section 43 will no doubt afford the public every necessary protection. It is thereby enacted that when the Lord Chancellor does not in his order for inquiry direct the return of a jury, but the Masters acting under the commission, upon consideration of the evidence before them, certify to the Lord Chancellor that in their opinion an inquiry before a jury is expedient, they are without further order to issue their precept to the sheriff, and to proceed in all respects as if the Lord Chancellor had directed the return of a jury in the first instance. We may well

* See Forms of Notices, General Orders in Lunacy, of 7th November, 1853. Nos. 8 and 10.

† 120 Hansard, 360.

suppose that the Masters, if only for their own protection, will under this provision always call for a jury in a doubtful case.

The inquiry, whether with or without a jury, is confined to the question whether or not the alleged lunatic is of unsound mind, and incapable of managing himself or his affairs at the time of the inquiry, except where the Lord Chancellor, under special circumstances, directs that there should be an inquiry from what time the alleged lunatic has been of unsound mind and incapable of managing himself or his affairs, or directs that there should be an inquiry whether or not the alleged lunatic was of unsound mind and incapable of managing himself and his affairs, at a previous time specified, and thenceforth down to the time of the inquiry, § 47. Lord Lyndhurst pointed out that no advantage was derived from carrying back the inquiry, and that it must often unavoidably lead to enormous expense;* and Lord St. Leonards said, "a large expense is now incurred by the inquiry at what time the party first became of unsound mind. The result of the inquiry on this point is in nine times out of ten of no value at all."†

Where the alleged lunatic resides out of the jurisdiction, the inquiry is to be before a jury, and "no further or other notice shall be necessary to be given to him than he would have been entitled to receive if this act had not been passed," § 45. With regard to notice, it has been said that the inquisition is an *ex parte* proceeding, and that therefore the alleged lunatic is not entitled to any notice, but in *ex parte* Cramer, a lunatic,‡ Lord Erskine said that "the party certainly must be present at the execution of the commission; it is his privilege." If this dictum be correct, it would seem that in ordinary cases, unless notice were given, the inquisition might be quashed. It very rarely happens that an inquiry takes place as to the lunacy of a person out of the jurisdiction.

The Lord Chancellor may from time to time, by order, regulate the number of jurors to be sworn, but so that every inquisition upon the oath of a jury be found by the oaths of twelve men at the least, § 46. The person executing an inquiry with a jury, while so employed, is to have all the like powers, authorities, and discretions as a judge of a court of record, § 48. The Lord Chancellor may still issue a commission specially, or may issue a commission directed to any person or persons in addition to the Masters in Lunacy, or one of them, if he shall upon any occasion deem it proper to do so, § 50.

Under the provisions of the statute 8 and 9 Vict., c. 100, § 94 and 95, if the Commissioners in Lunacy reported to the Lord Chancellor that they had reason to suppose that the property of any person detained as a lunatic was not duly protected, or that the income was not duly applied for his maintenance; or when any person had been detained as a lunatic for twelve months upon an order and certificates, the Lord Chancellor might direct that one of the Masters in Lunacy should personally examine such person, and take evidence, and report whether he was a lunatic; and on the Master reporting that such person was a lunatic, the Lord Chancellor might appoint a guardian of his person and a receiver of his estate, and might direct his income to be

* 120 Hansard, 352. † 123 Hansard, 184.

‡ 12 Ves. 445.

applied for his maintenance. But the proceedings under these provisions being less effectual for the protection of a lunatic and the management of his estate than proceedings under a commission, they have been discontinued by § 53 of the recent act; and now, by § 54, on the Commissioners in Lunacy reporting that the property of a person alleged to be a lunatic, or detained as such, but not found so by inquisition, is unprotected, or the income is not duly applied for his benefit, the Lord Chancellor may direct an inquiry, which is to be conducted in like manner as if a petition for an inquiry had been presented. It is well to have uniformity of procedure, especially as the expenses will be so much diminished by this act.

Many of the provisions included in § 55—97, regulating the proceedings after the inquisition, are new. Under § 59, where an affidavit is required for verifying all or some of the statements contained in a petition, state of facts, proposal, or other document, the affidavit may be annexed or underwritten thereto in the short form given in the third schedule, with such variations as the circumstances may require; and where the Taxing Master is of opinion that the form is applicable, the costs of such an affidavit only are to be allowed. This affidavit is to be made by the petitioner or other person bringing in the document, and is to be to the effect that so much thereof as relates to his own acts and deeds is true, and so much thereof as relates to the acts and deeds of any and every other person he believes to be true. This appears to be a very loose mode of taking evidence, but experience will show how far it can be made to answer.

The Masters in Lunacy instead of the Attorney-General now approve of the security to be given by the committee of the estate, § 62; and the approved committee may be permitted to transfer stock or pay money into court, instead of entering into a bond or recognizance with sureties, § 64.

Sec. 63 directs that the grant of custody be dispensed with, if her Majesty shall think fit to authorize the Lord Chancellor to make orders for the custody of the persons and estates of lunatics, without requiring a grant to pass under the great seal; and any such order will (as to the custody of the person immediately, and as to the custody of the estate upon the Master's certificate of completion of the committee's security) have the same force and validity as a grant under the great seal. The appointment of committees is now made by order instead of a grant under the great seal. Where several persons are appointed joint committees, the grant or order of custody may be extended so that, on the death or discharge of one or more of the committees, the others may continue to act without a fresh grant or order, § 66.

The Masters may receive and deliver out deeds or securities belonging to the lunatic, and authorize the payment or transfer into court of money or stock belonging to the lunatic, § 65.

The Masters may, without any order of reference, receive any proposal, and conduct any inquiry relating to the person or estate of the lunatic, and report thereon whenever they are of opinion that if application were made to the Lord Chancellor concerning the same, a reference would be made to the Masters, § 70; and this provision is extended by the general order, No. 14, so as to embrace any inquiry affecting the lunatic's property.

Subsequent sections provide for objections made by parties attending before the Master, and the allowance of the costs of such proceedings. Sections 75—81 relate to inquiries as to next of kin, dispensing with or limiting such inquiry when it may be inexpedient, and regulating their attendance on the proceedings; and by § 82 the Master may appoint a guardian to infant next of kin, for the purposes of the lunacy. The Masters may direct the time at which any proceeding shall be taken or continued before them, § 86; inquire into delays, § 87; and disallow unnecessary costs, § 88.

Sections 90—97 regulate the form and confirmation of reports. Objections to the report must be submitted to the Master in writing, and if insisted on after being disallowed, they must be brought forward in opposition to the confirmation of the report without petition; where there are no objections, or they are abandoned, the report is to be submitted to the Lord Chancellor for confirmation, without petition, and without the attendance of parties, unless the Master shall otherwise direct; and when submitted without petition, the report must contain the directions consequential on its confirmation, and the fiat of the Lord Chancellor on the report will give it the operation of an order.

This is not following the principle of the Joint-Stock Companies Winding-up Acts and the Irish Chancery Acts, as was suggested by Lord Lyndhurst; namely, that instead of the Master making a report (requiring the confirmation of the Lord Chancellor), he should in the first instance make the order, which should be binding and conclusive on all parties, if not appealed from to the Lord Chancellor within a given time. It may well be doubted whether the plan adopted will work satisfactorily in the case of reports to be submitted to the Lord Chancellor for confirmation without petition and without the attendance of the parties. It is desirable to save unnecessary expense, but it is more important that the business should be efficiently performed. Either the confirmation of the report by the judge will be a mere form, or he must have before him and read all the evidence upon which the report was made, and even then he would be in a worse position than the Master, for he would be without the assistance afforded by the attendance of the parties whose knowledge of the facts and circumstances under consideration must be of material aid in the decision of the particular matter in question. So that, if the system of reports is to be adhered to, the reports should still go to the Court on petition; for if the judge is to go through all the evidence, as he must do if the parties are not to attend him, he might as well dispense with the report, and himself make the order in the first instance. But experience will show whether this part of the new practice will work. In any case, we should prefer seeing the Masters working out the matters before them by orders subject to any proper power of appeal; it is a waste of judicial strength to let two officers be engaged in the work of one.

Reports are to be brought before the Lord Chancellor for confirmation by petition in each of the several cases specified in § 97. Orders are regulated by § 98—103. The visiting of Chancery lunatics is regulated by § 104—107.

Many of the provisions contained in § 108—147, relating to the management and administration of the estates of lunatics, are re-enacted from the statutes 11 Geo. 4 and 1 Wm. 4, c. 65, as to the admittance of lunatics to copyholds and payment of fines; the surrender of leases to which the lunatic is entitled, and acceptance of new leases; the sale, mortgage, or leasing of the lunatic's property; the transfer of stock, &c.; but there are also several new enactments of great importance. The powers conferred upon the Lord Chancellor by the 11 Geo. 4 and 1 Wm. 4, c. 65, as to the sale or mortgage of the lunatic's property for his benefit were very limited, and these powers were only slightly extended by the statute 15 and 16 Vict., c. 48 (an Act for the amendment of the law respecting the property of lunatics); but now, by the present act (§ 116 and following sections), the Lord Chancellor has almost absolute power over the lunatic's real as well as personal estate, whether in possession or in expectancy: by § 116, where it appears to the Lord Chancellor to be just and reasonable, or for the lunatic's benefit, he may order that any estate or interest of the lunatic in land or stock, either in possession, reversion, remainder, contingency, or expectancy, be sold or charged by way of mortgage, or otherwise disposed of, as may to him seem most expedient, for the purpose of raising money to be applied, and may accordingly order that the money when raised be applied, for or towards all or any of the purposes following:—

1. The payment of the lunatic's debts or engagements;
2. The discharge of any incumbrance on his estates;
3. The payment of any debt or expenditure incurred or made after inquisition, or authorized by the Lord Chancellor to be incurred or made for the lunatic's maintenance, or otherwise for his benefit;
4. The payment of or provision for the expenses of his future maintenance;
5. The payment of the costs of applying for, obtaining, and executing the inquiry, and of opposing the same;
6. The payment of the costs of any proceeding under or consequent on the inquisition, or incurred under the order of the Lord Chancellor; and
7. The payment of the costs of any such sale, mortgage, charge, or other disposition as is thereby authorized to be made.

Where the net amount or estimated value of the property of a lunatic does not exceed 500*l*., and it appears to be expedient that the same should be made available for his maintenance in a direct and inexpensive manner, and that it can be safely and properly done, the Lord Chancellor may, instead of proceeding to order a grant of the custody of the estate, order the property to be realized and paid to a relative of the lunatic, or such other person as he may think proper, to be by him applied for the maintenance of the lunatic at his discretion, or as the Lord Chancellor may direct, § 120. This appears to be an admirable provision; indeed, some proceeding of this description is really the only way of giving a lunatic possessed of small property the means of enjoying it, provided the Lord Chancellor's order be made in a *summary* mode without an *expensive* inquiry in the first instance as to the lunacy and other matters. The costs of ordinary proceedings would almost swallow it up. The following section (§ 121) also contains a useful provision: when there is reason to believe that the unsoundness of mind of any lunatic found so by inquisition is in its nature temporary, and will probably be soon removed, and it is expedient that *temporary* provision should be made for his maintenance or the maintenance of his family, and he has a sum of ready money available for the purpose, the Lord Chancellor may authorize its application for the temporary maintenance of the lunatic and his family.

The provisions of § 123 and 124 will save much expense in the cases to which they are applicable. Where a member of a co-partnership firm becomes lunatic, instead of the expensive process of a Chancery suit being necessary for winding-up the affairs of the partnership, it may now be dissolved, and the property may be disposed of under the order of the Lord Chancellor (§ 123). By § 124, where a lunatic is seised of or entitled to an undivided share of land, and it would be for his benefit or expedient that there should be a sale or partition, or where an exchange of the lunatic's land would be beneficial or expedient, the same may be effected under the order of the Lord Chancellor, instead of a special act of parliament being necessary to effectuate the object. This will be of great importance in the management of lunatics' estates; and it will also remove a cause of great hardship to those who are interested in real estate jointly with a lunatic. Sections 129—135 regulate the granting of leases, and confer new powers as to leases by lunatics having a limited estate in land and leases of mines.* Much inconvenience has arisen from the inability of a committee to execute a power vested in a lunatic either for his own benefit or for the benefit of others. This is now remedied by § 136, 137, 138, under which the Lord Chancellor may authorize the committee to execute a power vested in the lunatic for his own benefit, § 136; or a power vested in the lunatic in the character of a trustee or guardian, § 137; and where a committee under the foregoing provisions exercises, in the name of the lunatic, a power of appointing new trustees vested in the lunatic, the trustee so appointed will have the same rights and powers as he would have had if appointed by the Court of Chancery under the Trustee Act, or by decree in a suit, § 138.

With respect to the traverse of the inquisition, the provisions of the 6 Geo. 4, c. 53, are in substance re-enacted in § 148—151 of the new act, and the only new provision appears to be that "no person shall be admitted to traverse oftener than once," § 150; under this section the Lord Chancellor still has power to direct one or more new trials if he should be dissatisfied with the verdict returned upon a traverse. No alteration is made in the law as to the right of the person found

* By the 18th Vict. c. 13, "An Act to explain and amend the Lunacy Regulation Act, 1853," extended powers of granting leases, &c., are conferred upon tenants-in-tail.

lunatic to traverse the inquisition, so that the law on this point remains as it was settled *in re Cumming*.* Lord Hardwicke† and Lord Thurlow‡ were of opinion that the traverse was not matter of right; but Lord Rosslyn,§ Lord Eldon,|| Lord Cottenham,¶ and Lord Truro** held that the traverse was a matter of right. *In re Cumming* the question came before Lord St. Leonards and the Lords Justices Knight Bruce and Lord Cranworth in full court, and they were unanimously of opinion that the traverse was a matter of right, subject to such a control over the matter by the Court as may be necessary for the protection of the person and estate of the alleged lunatic; as, for instance, the ascertaining that the application is *bonâ fide*, and that the alleged lunatic, where he is the person applying, is competent to judge of what he is doing, and is really desirous that the traverse should issue. It was stated in this case that there did not appear to be any instance in which the traverse had been refused when applied for by the lunatic himself.

Both Lord Lyndhurst†† and Lord Campbell‡‡ were of opinion that the traverse should not be allowed as of right, and Lord Truro proposed to meet the point by having the question of lunacy, when disputed, tried in the first instance before the judge at the assizes. But, of course, in this latter case there would be the right to grant a new trial if, in the opinion of the judge before whom the issue was tried, or in the opinion of the Lord Chancellor, there were grounds for a new trial. Lord Truro stated that, during the last forty years there had not been a single instance of the verdict upon the trial of the traverse being contrary to the verdict upon the inquisition.§§

It is provided by § 152 that when any person has been found of unsound mind by inquisition, but the question of unsoundness of mind is disputed, and liberty to traverse has been applied for, and whether granted or not, and it appears to the Lord Chancellor to be for the lunatic's benefit, and also expedient that the inquisition should be superseded on terms and conditions, and subject to an arrangement respecting the lunatic's estate, he may, upon the consent of the lunatic and of the person entitled or claiming to traverse, and of such persons, if any, whose consent he may deem necessary, order the inquisition to be superseded on such terms and conditions to be fulfilled by the lunatic or such other person, and subject to such arrangement respecting the lunatic's estate, as he may under the circumstances of the case think proper, and may by the same or any other order direct the lunatic and any other persons, being consenting parties to the arrangement, to do such acts as may seem necessary or proper for securing the fulfilment of such terms and conditions and the completion of such arrangement.

By § 153 the Lord Chancellor, with the advice and assistance of the

* De Gex, M. and G. 537. † Ex parte Roberts, 3 Atk. 5.

‡ In re Fust, 1 Cox, 418.

§ Ex parte Wragg, 5 Ves. 450; and ex parte Ferne, 5 Ves. 832.

|| Ex parte Ward, 6 Ves. 579. ¶ In re Bridge, Cr. and P. 338.

** 1 De Gex, M. and G. 541, 551.

†† 120 Hansard, 358. ‡‡ 126 Hansard, 903. §§ 120 Hansard, 364.

Lords Justices, is empowered to make general orders for carrying into effect the provisions of the act, and regulating the form and mode of proceeding and the practice in lunacy. Under this provision general orders, 56 in number, were issued on the 7th November, 1853, whereby the previous general orders of the 27th October, 1842, and 15th April, 1844, are discharged. Orders 7—10 regulate the notice to be given to an alleged lunatic of the petition for inquiry; and he may within seven days after such notice demand that the inquiry be had before a jury. By other orders the jurisdiction of the Masters in Lunacy is extended; and for the purpose of saving repetition in orders in lunacy that may from time to time be made, of directions usually inserted therein, many of such usual directions are embodied in the new general orders, 34 to 56.

A glance at the 153 sections of the New Lunacy Regulation Act, and the 56 General Orders issued under its authority, would show that they effect many and valuable improvements both in the law and the practice, and we feel bound to express our sense of the great praise which is due to Lord St. Leonards for the valuable improvements he has been able to effect in this branch of the law. The dispensing with the special commission in each case, and the jury in certain cases,* the abolition of the grant, and the enlarged jurisdiction of the Masters, considerably reduces the expense of proceedings; the abolition of nearly all fees, and the substitution of a graduated percentage upon the incomes of lunatics, is also a great relief to small estates; and as regards the amount of percentage paid on the large incomes, it will practically be an *ad valorem* payment, not for the administration of justice, but for administering the lunatic's property. The extension of the Lord Chancellor's power of dealing with the lunatic's real estate, as well as his property generally, is also highly important, and many other minor changes are valuable; but at the same time we think that the practice may be simplified to a much greater extent, and the expense so reduced as to allow the property of every case of a chronic and incurable lunatic, who has property in his own right, and is not merely dependent upon eleemosynary support of relatives or friends, to be brought within the jurisdiction of the Lord Chancellor, and under his authority, so that his property may be legally administered for his benefit. We think something similar to Lord Lyndhurst's bill would be a decided improvement on the recent act. Instead of reports requiring an order to give it operation, orders would be made at once, which would not only lead to a further saving of expense, but also to increased expedition in the despatch of business. There would be the right of appeal whenever it was desired. The matters requiring the decision of the Master or the Judge are generally not points of law, they are administrative matters, no doubt involving points of delicacy and importance; but all that is necessary is to obtain the consideration by a person of competent experience and authority, and his direction for carrying into

* The friends of lunatics have often been most reluctant to resort to legal proceedings, owing to the painful exposure of a public exhibition of their relative before a jury. The privacy with which an ordinary inquiry is now conducted may well be included among the great benefits conferred by this Act.

effect, according to law and the rules of the court, the wishes and suggestions of the family and friends of a lunatic, with regard to his care and the management of his property.

We subjoin the principal clauses of Lord Lyndhurst's bill:—

SECT. I.—After inquisition found, it shall be lawful for the Masters in Lunacy to conduct all such inquiries, as to the care, custody, and management of the person and property, and to make such orders relating thereto, and for payment of money into court, or for sale or mortgage of real or personal estates, or for leases of real estate, and generally to make such orders, and give such directions in relation to the persons and properties of lunatics, as may from time to time seem to such Masters necessary and proper; and for the purposes aforesaid the Masters shall have (subject to the restrictions and regulations herein provided) the same jurisdiction, authority, and discretion, and the same power to make orders, and otherwise to act in and about the matter, as the Lord Chancellor or the Masters in Lunacy could have exercised or done in relation thereto, according to the ordinary practice of the Lord Chancellor in lunacy: Provided always, that it shall be lawful for the Lord Chancellor, if he shall see fit, by order, to direct that the matter of any person found lunatic by inquisition shall be excepted from the provisions of this Act, so far as they give the said Masters the same jurisdiction, authority, and discretion as the Lord Chancellor, and the same power to make orders, and otherwise to act in and about such matter, and that thereupon the proceedings relating to such matter shall be conducted according to the ordinary practice in matters in lunacy before the passing of this Act.

SECT. II.—No order of the Masters under this Act shall require confirmation, except when such order is made subject to the opinion of the court, or is a special report, as hereinafter provided, but every such order shall have the same authority and effect, and shall be binding on the same persons, companies, and bodies, and may be enforced by the same, or any such process, as if the same had been made by the Lord Chancellor, or as may be directed by any general orders to be made in that behalf as hereinafter directed.

SECT. III.—The Masters shall have power, if they think fit, to make a special report in any matter in which they shall proceed under the authority of this Act, or upon any question or matter arising in the proceedings thereupon, or to make any order, subject to the opinion of the court, to the intent that the opinion of the court may be taken on the subject of such order, and such special report or order shall be brought before the Lord Chancellor upon motion; and on the hearing, such report or order respectively of the Master shall be confirmed, discharged, or varied, or such directions shall be given as to the Lord Chancellor may seem just.

SECT. V.—An appeal shall lie to the Lord Chancellor, upon motion, from or against all orders, directions, and other proceedings of or before the Masters under this Act; provided that such appeal shall be made within fourteen days from the time when such order, direction, or other proceeding shall be made or taken, or such further time as the Masters shall by order made in the matter allow.

### IMPORTANT MEDICO-LEGAL TRIAL—THE PLEA OF INSANITY.

Robert Hancock (41, n) was indicted for the murder of his wife, at Northam, in August last, and was tried at the Winter Assizes, Dec. 15, 1855. The prisoner, on appearing at the bar, was visibly affected, and on the charge being read, sobbed bitterly, and was scarcely able to utter "Not Guilty."

The prisoner is a mild-looking man, of the labouring class. He is about 40 years of age, and has light hair and whiskers. He was attired in a fustian jacket and dark trowsers. Throughout the trial, he was often affected to tears, and his restless eye and perturbed manner showed the anxious feelings which were working within him.

Mr. Karslake and Mr. Bere prosecuted; and Mr. Cox defended the prisoner.

Mr. Karslake, addressing the jury, said the investigation upon which they were about to enter was one which demanded their very serious attention. The charge against the prisoner at the bar was no less a charge than that of murder upon the person of his wife. He (the learned counsel) was glad that they (the jury) had been chosen from a part of the county far removed from that in which the occurrence took place, because he would not have them brought into the jury-box in any way prejudiced by anything they had heard outside this court; and if any of them had preconceived, or prejudged, he hoped they would dismiss all such prejudices from their minds, and listen only to the evidence which would be laid before them. Having said this much, he thought he would best discharge his duty by detailing the particular acts and particular circumstances which took place prior to the decease of the unfortunate woman as to whose death it was their duty to inquire. Robert Hancock, the prisoner, had been married to his wife more than twenty years. There were two children by the marriage, but neither of the children were living with Hancock at the time of the death of the wife. He and his wife were living together at a small cottage at Northam, near Appledore, a town or village situated upon the sea, at the mouth of the estuaries of the rivers Torridge and Taw. He believed that for about seventeen years the prisoner and his wife had been living at Northam, and during the greater part of that time they lived in peace and harmony, and behaved to each other as man and wife ought to do. But for some little time before the dreadful occurrence, which took place on the 1st of August last, bickerings and quarrels existed between man and wife, the result, in a great measure, of a jealous feeling entertained by the prisoner toward his wife, which was constantly showing itself in observations, revilings, and quarrels which took place. He (the learned counsel) did not know whether the prisoner had any cause of jealousy or not, but certain it was, from many observations they would hear detailed, that he had frequently charged her with having been too intimate with a man named Punchard, and she, he regretted to say, did not give that contradiction to the charge which was made against her which she ought, but rather fostered the charge, and represented herself, at all events, as having been intimate with Punchard. At home this was a constant cause of irritation, and a constant quarrel; and at last, on the 1st of August, that dreadful occurrence took place which formed the subject, or rather, which had led to the investigation which they were now to enter upon. It seemed—and without going into the quarrels specifically, he would take up the case as regarded the facts of it, upon the 1st of August, the day on which the unfortunate woman was killed,—that about three o'clock in the afternoon of that day, the prisoner was with his wife in the house of a woman named Hele, and while there, one of those violent quarrels took place between them, and expressions were used by the prisoner towards his wife which he would not repeat, but leave to the witness Hele. The prisoner was one of the labouring class, and his wife also had been in the habit of labouring in the fields: and during what was called the lime season, the time of year at which vessels from the coast of Wales came to Appledore with lime, both of them were frequently in the habit of working, heaving limestone, that was, discharging it from the vessels. The quarrel which, as he told them, took place on the afternoon of Wednesday, the 1st of August, appeared to have been to a certain extent, indeed very considerably, quieted up between the man and wife, and on the evening of that day they both proceeded to Appledore, for the purpose of getting a job at lime heaving. It appeared that when they got there, there were a sufficient number of persons already engaged, and in consequence they could not obtain any work upon that occasion. It appeared that the prisoner and his wife came home together as far as Northam. They came home in company, and, as witnesses who would be called before them would tell them, apparently at that time on good terms, the prisoner doing some little act of civility to his wife, in carrying a bundle of rough clothes which she had taken with her to put on in prospect of getting work at lime heaving. They were then in company, and they were never seen together again; and the wife was never seen alive after that time. About nine o'clock on the same evening, the prisoner was seen to light his pipe at the house of a woman named Jane Saltern, at Northam, and at eleven o'clock he was seen going in the direction from his house, in a lane called Back Lane, in the village of Northam. The prisoner was not seen again till two days afterwards. On the morning of the following day, Thursday, the 2nd of August, some suspicions being entertained, in consequence of a child which had been taken in to nurse being heard to cry in the house of the prisoner, the woman, Jane Saltern, opened the up-stair room in the house, and she there found upon the bed the body of Phillipa Hancock, dead. In company with others, examination was made of the person of Phillipa Hancock, and very shortly afterwards, it was found that beyond all doubt death was caused by a most severe blow inflicted upon the left temple and the throat being cut. The pillow and bed-clothes were covered with blood. The hammer by which this deed was committed was lying upon the pillow, and there could be no question whatever in this case that the wounds which appeared on the unfortunate woman's person were not self-inflicted, but feloniously inflicted upon her. Search was made for the prisoner, who was not in the house, and he was not found during the next day, but on the morning of the following day, Friday, the 3rd of August, he was seen in a tallet, which the jury would know was a loft above a stable. He went from there, and went across some fields, and afterwards gave himself up to a man named Dennis, his brother-in-law, who was seeking for him in the village of Appledore. He (the learned counsel) had told them the facts which would be presented before them in this case by witnesses, and it would be material for them to listen attentively to these facts, because, in all probability, they would raise a very strong presumption that the hand which committed the act upon the person of Phillipa Hancock was the hand of the prisoner at the bar, and the more so when he told them that he gave up a razor, which, no doubt, was such an instrument as caused the wound in the throat, to Dennis and the constable. There was some blood upon the hammer found on the pillow, and blood upon the blade of the razor. He (the learned counsel) had stated to them what would be spoken to by witnesses who had knowledge of the facts of the case, and probably it would be better that he should not detain them by statements made by the prisoner subsequently, as they would be laid before them by the witnesses to whom they were made. They would find by these statements the fullest admission of the fact that by his hand that murder was committed, and that it was committed first by blows from a hammer, and afterwards by the throat being cut by the razor. These were the facts which would be laid before them, and he believed they must bring it home to the prisoner as the man who murdered his wife. His friend Mr. Cox was for the defence, and he was at a loss to understand what defence he should make, but he (the learned counsel) would have an opportunity, if he called witnesses for the defence, of again addressing them on the facts. The learned counsel then called the following evidence:—

*Jane Saltern*—I am the wife of Henry Saltern, and live at Northam. I know Robert Hancock, the prisoner, and I knew his wife. They lived at Northam, near me. I have lived in the village all the days of my life, and the prisoner and his wife have lived there 16 or 17 years. I remember Wednesday, the 1st of August last. Prisoner came to my house at nine o'clock that night to light his pipe. I lived next door to him. I was called by Mary Bere on the following morning, and I went to Hancock's house—the door was unlocked, and I went in. I saw the prisoner's shoes and his wife's shoes at the bottom of the stairs, and then I went to the foot of the stairs and called upon them both. There was no answer.

By the JUDGE—A little child, about three years of age, lived in the same house with the prisoner and his wife, and no one else.

*Examination continued*—I then went up stairs, and saw the deceased, Phillipa Hancock, lying on her right side on the bed. I saw the marks of a blow on the side of her head. She had a cap on, and there was blood on the left side of her head. I was so frightened that I ran downstairs and hollaoed "murder." This was about half-past one o'clock in the day. I saw George Labbitt, and said to him—"Phillipa Hancock is murdered." Before this the prisoner used to throw-up (or accuse) to her, Punchard, and threaten her life. He was a mason, and living at Northam. The prisoner and his wife had been next-door neighbours to me about six months, and during that time there had been frequent quarrels between them. I have heard the prisoner say he would murder her; he said so in my house, and in the presence of his wife, about a week before the 1st of August, when the crime was committed. I heard him say that he should not be easy until he had killed her, and he would then kill himself afterwards. I never saw him in a passion in my life; it always seemed as if there was something out of the way with him.

His LORDSHIP—What do you mean by that?

*Witness*—I think there was something the matter more than common, because he put out such naughty words.

Mr. BERE—To whom did he use these words?

*Witness*—To his wife. I cannot repeat what he said—they were bad words such as I would not use.

His LORDSHIP—Were they unchaste words, imputing to her that she was a bad woman?

*Witness*—Yes, sir; that's what he meant, with William Punchard.

His LORDSHIP—Did he use coarse and vulgar expressions?

*Witness*—Bad words. He seemed not to be in a passion, but spoke them as if he meant what he said.

*Cross-examined*—She had known him for many years; and it was about two years ago that she heard he was in the habit of using these words. She only heard them from about six months ago. She did not know he was so bad before that—it was different from what she had ever heard of him. He was always very kind except in this matter with his wife. Punchard lived at the head of the street, not many yards from the prisoner's house. I have heard other people use hard words, but they were not like the way he did it. From the odd way in which he did it, I thought there was something wrong about it.

By the JUDGE—I did not think he was in his right mind in using such bad words to his wife.

His LORDSHIP—Do you mean to say that you thought he was under a mistake and from his mind being wrong he imagined these things?

*Witness*—I thought there was something more than common about him and I was struck with his making these charges against his wife. I thought he was bringing a false charge against her. I did not think there was any truth in it, nor that there was any cause for his jealousy. I was on intimate terms with his wife, and never saw anything going on wrong. I never saw Punchard go into the house, nor never saw anything going on wrong between him and deceased.

*Re-examined*—I had heard deceased say to prisoner, "Why should you throw it up to me, when I bain't deserving of it." At one time, and before this happened, Punchard lived in a house next but one to the prisoner, and after that prisoner's wife came to live next door to me. I have seen prisoner, deceased, and Punchard together. These were the only times I ever heard prisoner use bad words when he was accusing his wife.

*George Labbitt*—In August last I lived near Hancock—two houses and a garden separated us. I had known prisoner and his wife about seven years. I remember being told by Jane Saltern of something having occurred. This was about half-past one o'clock. I went to Hancock's house, which consists of three rooms, two up stairs and one down. I went up stairs, and saw the deceased lying on the bed on the floor; there was no bedstead. She was lying on her right side; her legs were across, and one leg was partly uncovered. The bed clothes covered the top of her person. I saw blood on the side of her head and the strings of the cap were saturated with blood. Her right arm extended across the bed. A hammer lay on the pillow by the side of her head. There was no razor; I did not look for any. The deceased was lying as though she was perfectly composed—her left arm being across her stomach. I did not examine her wounds; Philip Dennis came before I left. I went down stairs and brought Willis with me, and I then left the body, which was quite cold and stiff. Dr. Pridham came to the house whilst I was away. I was absent for a quarter of an hour and when I returned there were several persons there, among them being Dr. Pratt and his son. The latter handed the hammer to his father, which was the same as I had previously seen. I put a screw over the latch, so that the door could not be opened without removing the screw, and the hammer was left in the room. The prisoner was not in the house on the day in question. I made search for him about the outskirts of Northam, but could not find him. I have heard the prisoner and his wife quarrelling as I passed the door, but nothing more.

*Cross-examined*—It was lately that I heard them quarrelling, since they removed to the house next to the first witness.

*Philip Dennis*—Philippa Hancock was my sister. I heard from Jane Saltern, about half-past one o'clock on the 1st of August, of her being dead. When I went to the house, many people were in the bed-room. I saw my sister lying in a pool of blood, with the hammer on the pillow by her forehead. It was a large hammer. Mr. Pridham, surgeon, came afterwards, and he sent for Mr. Pratt. I came away with the rest of the people, when the door was screwed up. There was a little child in the house, which my sister took care of, but it was not her child. My brother-in-law and sister had been married 20 years, and lived in Northam 17. They had two children, one 12, and the other 16. They both lived in service. Hancock was not in the house when I was there. I found the remains of raspberry pie on the table, with a cup of cream and two plates, just as they had been used, in the down-stairs room. I and others made inquiries for Hancock in the afternoon, but I could not find him. On the following Friday I was at work at Knapp, a half a mile from Northam. I saw people going across a wheat-field, and saw a man going over a bank. I followed, and went in the direction I supposed him to have taken. I afterwards saw

Hancock; he came up to me from a ditch, out of a brake. Shortly after that, a man named Parkhouse came up, and I had some conversation with the prisoner. I said, "Oh! Robert, what have you done?" I did not hold out any threat to him. He said, "Oh! Philip, I have killed your sister." He then took hold of my hand, and said, "It is that rogue Punchard who has caused me to do it." [Here the prisoner, who had hitherto assumed an attitude of prayer, took a little book from his pocket, which appeared to be a Catechism, and commenced perusing it very devoutly.] I asked him what he had done it with, and he said he had done it with the hammer and the razor. I asked him where the razor was. He said, "I have it in my pocket; but I shall not give it to you, because it is bloody." I did not examine his pocket. This was said before I joined Parkhouse, and when the prisoner first came out from the brake. I asked him what time he had done it, and he said, "About half-past nine." I then saw Parkhouse and others assembling together in a field, and I and Hancock went up to them. I then repeated my question, before these persons, as to the time he committed the crime, and he told me half-past nine. We then all went on to Northam, where we met Braund, the constable, who took him into custody. Braund asked him why he had done it, and if he had a knife. Prisoner said he had no knife, and gave him up the razor. I saw the razor; there was some blood on it. I afterwards went to the lock-up, where Braund and the prisoner were. The latter was, I think, locking the door. I asked prisoner again what could possess him to do it. He said, "I can't tell you—I've a done it." I said, "What could have possessed you to do it, when you came from Appledore together in the presence of James Dymond, and appeared to be comfortable?" I added, "When you both ate supper together, which was raspberry pie." I said, "We found the pie and cream with it." Prisoner said, "Yes, there was some cream, for I fetched it myself. Then we ate supper together. I thought to go to bed comfortable, but she would not let me come into bed. I said to her, 'If you will not let me come into bed, I will go again.' I then went over to William Cleverdon's, and got a half an ounce of tobacco." Thomas Braund was then present, and said, "Cleverdon says it was on Thursday morning at nine o'clock that you had the tobacco." He said, "No; Thomas Cleverdon's mistaken; it was Wednesday night I had the tobacco. When I went in at the door, there was a little maid coming out with a loaf. William Cleverdon tended me with the tobacco himself. I filled my pipe in his own house. I went towards home, and lighted my pipe at Jane Saltern's. I smoked my pipe at the corner of the chapel, where I spoke to Thomas Harris. I smoked out my pipe, and went into my own house. I went up stairs to go to bed, and took the hammer and razor with me. I asked her then if I should come into bed, and her said, 'No, you shall not come into bed.'" I said to him I believed she was asleep. He said, "Her was not asleep." I believe he said, "I asked her the third time if I should come into bed: then I gave her a light knock on the head," but he did not say what with. Her cried out "Oh! Robert, don't hurt me." He said, "I fancied I saw a little blood there; I thought I might as well go through it as not. I rose my hand and struck her very lusty, and the blood gushed out. After that I threw the hammer directly down. Then I cut her throat—I thought I would put her out of misery as soon as I could. I remained in the house until about eleven," but he did not say whether he meant night or morning. He then said, "I left my house and went up Back-lane. I met three men up at the corner of Burrough" (which is a farm-house near the Burroughs). I said, "Yes, Robert, if it's true, you were met there." "Then I went down towards Cleveland (which is a gentleman's house), and crossed over Thomas Bellem's field. I came out by Crosse's, and went down over Mr. Partridge's field, and came out again by Holywell. I crossed again over Mr. Partridge's field, and came towards Lewis' Hill. Then I intended to have come to see you, and tell you what I had done; but my heart failed me, and I could not come to you. If I could have come to you, I should have cut my own throat. I went into Perkins' grass-field, and up over his turnip-field. Then I thought I could have come to you, but my heart failed me. Then I went into my own house, and lighted a candle. I went up stairs and looked at my wife, whom I had killed. [Here the prisoner laid down his book, put his handkerchief to his eyes, and wept bitterly.] Then I felt very sorry, but it was too late. I went down again, and blew out the candle. I closed the door, and left the house. I went into Mrs. Balsdon's house, and waited there for Mary Hele until her return from heaving limestone, for I wanted to tell her what I did want to tell you. I waited there to see her, to desire her to tell you to take care of the children. When Mary Hele came up, Thomas Wilkey was with her; and because he was with her, I couldn't tell her what I wanted to tell her. I left the court, and went down the road towards the barn, where Parkhouse saw me, and just as I came there I heard the clock strike two." Thomas Braund then said to prisoner, "You were at Appledore yesterday morning; John Tucker saw you there, with an umbrella in your hand." Prisoner said, "On Wednesday morning I was there; when John Tucker saw me there, I had an umbrella in my hand, and my wife was there with me. I went into a shop to buy a few things for my daughter, which is now at home in a paper in a box. It is not made up, but I hope my daughter will have it." I suppose he meant some dress. Prisoner said, "I love my daughter, and her loves me." [Here the prisoner sobbed aloud.] I said, "How can you say you love her, when you said the other day that when she came home again, she would come home to her mother's funeral?" He said, "Yes, Philip, I did say so, and now it is so." Prisoner's daughter had related the above at the house where she lived, and I heard of it. Prisoner begged me to take care of his children, as they were those of my own sister. That was all that took place in the lock-up on Friday, the 3rd of August, and I had no further conversation with him. I had for some time lived in the neighbourhood of Northam, and frequently saw prisoner and deceased. When first they were married, they lived very comfortably; but she was rather violent in her temper. The quarrels began about two years ago; I have been present and heard them quarrelling; it was about twelve months ago that I first heard of the cause of the quarrel. I heard him throw up to her about Punchard, saying that he had been with his wife. She said, "If I have been with Punchard, I will go with him again." I have heard these quarrels many times. I have never seen Punchard with her.

By the Judge—Punchard is a middle-aged man—about forty. My sister was about the same age.

*Examination continued*—When the prisoner lived neighbours with Punchard, deceased used to go to his house, but I never knew any harm of her. I never saw anything particular between her and Punchard. Prisoner wanted her to leave off frequenting Punchard's house, and I have heard her say—"I will go to Punchard's house when I like—for Punchard's a man, and that's more than you are." I should think this must have occurred more than eight months before. I have heard her say, "If I had sixpence, I would give Punchard threepence." I have heard these sort of quarrels going on twelve months before her death. When she said if she had got sixpence she would give Punchard threepence of it, prisoner asked deceased to leave the house and take one in another part of the village, or either go over to Wales, and he would never say anything more about Punchard. His wife said, "I will not leave the house; as long as I am spared I will live neighbours by Punchard." Just after this prisoner left his wife, intending to go to Cornwall. He bided away from the Tuesday until Sunday, and then returned. The neighbours joked him for coming back, and he said, "The reason of it was—the love he bore to his children." Then he lived with his wife till Saturday, 3rd of February, constantly quarrelling; and on that day he came to my house with several of his working tools. He said he had left his wife, and couldn't live with her. On the same evening he returned to his house for a wheelbarrow, saying he would sell it, take the money, and go to Wales. He remained at my house seven weeks. During that time his wife lived neighbours with Punchard. When he went back for the wheelbarrow, he and his wife had a great quarrel, and several neighbours interfered. At the expiration of the seven weeks he went back with his wife again, as she had removed to the house where she died. In the quarrel about the wheelbarrow, prisoner threatened to kill Punchard; and I believe that was the reason for her removing. My sister was a strong, powerful woman, and used to work at heaving the limestones from the barges at Appledore. Prisoner used to do the same. I remember on one occasion returning from Appledore with the prisoner, Punchard, and Punchard's wife. The prisoner said to his wife he never would hurt Punchard; to which she replied, "No, Robert, you never had occasion to say what you have said"—but what that was I did not hear. Prisoner said, "Anne, it is true; what I have said he is guilty of." Punchard heard these observations, but made no reply.

*Cross-examined*—Punchard's wife was very good friends with my sister, and when she went into Punchard's house it was to see Mrs. Punchard. Mrs. Punchard was never jealous of my sister, that I heard of. I never heard the neighbours taunting the prisoner. I remember his being taken up, and brought before Mr. Gould. I think it about two years ago when prisoner began to get jealous of Punchard. He was the same man as before, except on this subject. I never teased him about it. Prisoner told me he had seen Punchard having intercourse with his wife, and he described where it took place. He told me he could have touched them. He said he had sat on a neighbour's house, and seen them at his own back door. No doubt if he had been at the top of the house he could have seen it. That was in February. When he said he could have touched them was just before this. He said if he had touched them he must have killed one or the other; but he thought if he let them alone, and told his wife all that had taken place, she might leave it off and be better.

By the Judge—On the occasion when he was so near that he could have touched them, he told his wife that he intended to go to Bideford, but he did not intend to go. He said at one time it was at his back door on a heap of dung. I had never seen anything between my sister and Punchard to lead me to believe that there was any improper connexion between them.

*Thomas Braund*—I am constable of Northam, and knew Philippa Hancock. At half-past two, having heard that Hancock had killed his wife, I went to his house and went up stairs, and found the body of Philippa Hancock on the bed. Dr. Pratt and his son were present. Dr. Pratt's son gave me a hammer. I saw a little blood on the handle of it. I kept the hammer for about two hours, and then laid it down beside the body. I went next morning in search of Hancock; I saw Hancock with Philip Dennis and John Parker. I then took him into custody. I said to him, "Oh, Robert!" He replied, "I have done it." I then asked, "Have you a knife about you?" and I asked him where the razor was, and he gave it me. I produce it. There was blood on the razor when I got it. I then took him to the lock-up house, and was present during part of the conversation which took place between Dennis and prisoner. [The razor was an old white-handled one, very rusty, and was handed round among the jury. At sight of it prisoner wept very much.] Last April I took the prisoner into custody, at the request of Mary Anne Punchard and Mary Anne Hele, on account of a quarrel with his wife. I took him into custody in his own house. He had his shirt-collar unbuttoned, and his jacket, waistcoat, and neckerchief were off. It was about ten o'clock at night. His wife and several other persons were present. I heard him say, "I will have murder in this house this night." I said, "No, Robert, I will take care of that." All this time he was walking about the house in an excited state. He said to his wife.

"You know you are guilty of what I accuse you." The wife made no answer, but sat crying. I then took him away to a beer-shop, and kept him there that night. In the morning his wife came and offered to give him some breakfast. He refused to take the breakfast, and said, "I'll have no more breakfast from you: you are going to send me away; send me to where I may never come back again." During the night he said to me, "People say I am mazed."

By the Court—I never observed him out of his mind.

*Examination continued*—He said, "I am no more mazed than those who say so." I took him to Mr. Gould, the magistrate, but no one appeared against him, and he was discharged. Mr. Gould told him to live on better terms with his wife. That was all he said then.

*Cross-examined*—I did not see him search or call for a razor in this house. I did not see him tear his hair. The wife said nothing about sending him to an asylum. She said nothing to me at all.

Counsel—What was meant by his saying, "You are going to send me away?"

*Witness*—I don't know. Nothing was said about a lunatic asylum in my presence.

*John Mill*, constable of Northam—I heard on the 2nd of August of the death of Mrs. Hancock, and went immediately to the house. I saw Mr. Pridham and Dennis there. I saw the hammer lying at the head of the woman. I was present at the inquest, and, after the inquest, took possession of the hammer, which was given me by a woman who was present. I produce the hammer.

[The hammer was a little thick, clubbish instrument, but very heavy—such as is used by blacksmiths for making nails.]

*Thomas L. Pridham*, surgeon at Bideford—On Thursday, 2nd of August, I was at Northam visiting a patient. About the middle of the day, I heard of a woman having been found dead, and went directly to see the body. It was the body of Mrs. Hancock. It was a little before two. I found the body on the bed quite cold. The body was lying partly on the right side, the right hand stretched across the bed, the left hand across the chest; the left hand and arm much besmeared with blood. This gave me the idea that a struggle had taken place. The legs were lying in an easy position, as of a person asleep. I felt parts of the body and as far as I could ascertain there was no warmth. I should think the body must have been dead eight or ten hours. I observed the state of the head, and saw considerable injury had been inflicted on the left temple, and a transverse wound inflicted on the throat, about three inches below the chin. This wound was about three inches in length. A considerable quantity of blood saturated the pillow and adjacent clothes which appeared to have flowed from this wound. I could not say whether the wound was inflicted during life or afterwards; if after death, it must have been immediately, and before the circulation had ceased. I sent for Mr. Pratt, and to the coroner. Next day an inquest was held and I made, in company with young Mr. Pratt, a *post mortem* examination. After shaving off the hair we discovered a considerable injury on the left temple. We then dissected the scalp and found a great quantity of blood between the scalp and the pericranium. The temple muscles were also injured; and when these were dissected off it was found that two severe blows had been inflicted on the skull, one corresponding with one of the external wounds. I then compared the hammer with the wound and found it to correspond. The other blow had also fractured the skull and was such a blow as might have been caused by the hammer. The one blow appeared much heavier than the other. In order to ascertain the extent of injury done to the brain the upper part of the skull was removed, when it appeared that something had penetrated the brain to the depth of an inch-and-half or more. It did not appear to be produced by such a large instrument as the hammer, but might have been by a splinter of a bone, though no bone was found. That blow would most decidedly have caused death. The wound in the throat measured three inches in a transverse direction, about three inches from the chin. The skin was divided and the carotid gland was divided. The windpipe was severed, and the instrument had gone so far that it entered the substance surrounding the spine at the neck. It had penetrated about half-an-inch into this substance. I should think the blows in the head were the cause of death. It is possible the woman might have lived had there been no wounds but those in the throat. I think the wound would have been produced by such an instrument as that razor.

*Wm. Pratt*, surgeon, of Northam. I assisted Mr. Pridham in making the *post mortem* examination, and having heard his evidence, I agree with it.

*John Parker*—I live at Northam, and work for Mr. Pratt, at Knap Farm. I was on the farm on the morning of the 5th of August, and went into the barn. I saw a man lying in the tallet, whom I found afterwards was the prisoner at the bar.

*Mary Ann Hele*—I live near Robert Hancock's, at Northam. On the afternoon of Wednesday, 1st of August, Hancock and his wife were in my house; they came about two o'clock. A quarrel took place between them. He kept throwing up about William Punchard. She did not make much reply, but he appeared to be in a great rage. He threatened he would kill her if she did not mend herself better about Punchard. She made no answer, but sat very solemn in the window. Afterwards she said to him he had threatened to kill her so many times, she would rather be dead than alive. She said to him, "You have threatened to kill me day and night so many times with a razor or a hammer, why don't you do it? and then I should be out of my fright." He got into such a rage that I desired him to go out of doors. He went to go out of doors, and as he went he looked over to where she was sitting, and said, "perhaps it might be quicker than she thought of." She said nothing, but looked to him and smiled, and that raised his passion more, for he thought she was laughing at him. He returned in two minutes, still in a great rage, and this quarrelling continued till about three o'clock. He thought when he came in that she was talking about him, but I said—"She is not." I remonstrated with him on what he had threatened to do, and said I was talking to her not to say anything about him to aggravate him. I said—"Consider the consequence to her soul, if this dreadful deed is done which you have been threatening." He said he intended to kill she, and to gulph her into the lowest pit of hell, when he would follow her. He intended to kill himself quickly after he had killed she. That is all that passed. I offered them to stay in the house, as I had to go out, and they at first agreed. I told them I would be back at four o'clock. Then he said the vessels were come to which we were accustomed to go stone heaving. Then she said she would go to her own house, and get the kettle boiled by the time I come back. She went to her own house, and he went with her. I returned in the afternoon about four o'clock, and between five and six in the evening went, with the prisoner and his wife, to Appledore, for the purpose of stone heaving. They appeared, in going, very comfortable, considering the quarrel they had. They could not get any employment, but I got some heaving, and did not see them afterwards. I know they had quarrels frequently, he ill-used his wife very much.

*Cross-examined*—I have known them married for seventeen years, and they appeared comfortable till within about six months before the murder. I heard of Punchard about fifteen months previously.

By the Court—I thought the prisoner never had any cause to be jealous of Punchard. It was a delusion, and more when he had his drink than at other times. I never knew him to be out of his mind on other subjects, in any respect.

*Cross-examination continued*—The prisoner gave me particular cases, but I thought it was a delusion. He was so filled with this idea, that he neither knew or cared about what he did.

*Re-examined*—I have heard his wife talk about Punchard. She said—"Since you have said so much about Punchard, if I have been with him, I will go with him again." I have heard her tease him in that way. She did not deny it, but teased him.

*James Dymond*—I am a labourer, and live at Northam. I knew Hancock and his wife very well. I saw them on Wednesday evening, the 1st of August. I came from Appledore with them, which we left about half-past six, arriving at Northam at a quarter after seven o'clock. They appeared then to be on very good terms. Prisoner's wife carried a bundle, which he, in a kindly way, attempted to take from her. He appeared to be perfectly sober when I left him. I have known Hancock upwards of fifteen or sixteen years.

His Lordship.—What was your opinion of the state of his mind?

*Witness*—I never saw anything out of the way in the man. I had some conversation with prisoner previous to this time. We were coming back from labour, and he began to tell about Punchard. He said he had every reason to believe that Punchard and his wife was great; and said he would kill her. I asked of him if he knew the consequences of it? You would be hanged; and her would be killed; and there would be an end of both of 'ee. He said he didn't care; he would have his revenge.

*Cross-examined*—Before this he always understood he was a very good fellow. He was not a violent man. He had not observed any change in him during the last two years. He was rather violent when speaking of Punchard.

By the Judge.—I never knew him irrational, or out of the way, on any other subject except Punchard.

*Thomas Taylor*—I am a tailor, living at Northam, and occasionally work at heaving lime stones from the vessels. I had been doing so on the evening of 1st of August. I returned to Northam with James Hearn and Lock. I arrived at Northam about ten or half-past ten at night. On my way home I passed down Bank-lane, and met Hancock, who was coming in the direction from his house. I wished him good night.

This was the case for the prosecution, and the court adjourned for a quarter of an hour. On resuming,

Mr. Cox rose, amid hushed attention, to address the jury for the defence. He said, if their anxiety at all approached his, it would be with something like a feeling amounting to awe that they would approach the decision of this case. The life of a fellow-creature was entrusted to their hands; for he was charged with a crime, the facts of which were undisputed. The question which they had to determine was, Is he a responsible being—was he acting under circumstances which made him answerable to his country for the crime which he had committed? That was really the question they would have to determine. That the unhappy man deprived his wretched wife of her life, there was no doubt; he (the learned counsel) had not attempted to throw any doubt or difficulty about the proofs in the case, for the man himself had confessed it almost immediately after it happened. He admitted candidly and frankly, almost to the first person he saw, that he had done it, and how he had done it; and now it remained for them, after they had heard the remarks from him, and with the assistance of his Lordship, to say whether the act was done with a full knowledge that he was committing a crime with a full mastery over himself, knowing what he was doing, knowing that he was responsible for what he was doing, and having the power of self-guidance, and being able to restrain the passion that was moving him to the act. He (the learned counsel) should submit to them that he had not, but that he was acting under a diseased mind, and that he was insane. He believed that after they had fully and carefully considered all the circumstances surrounding this case, and the conduct of the man both before and after the act, that they would come to the conclusion that he was not responsible for this act. By such a verdict they would not release him—a dangerous man—upon the country, but they would transfer him to an asylum for the rest of his life; they would be placing him where it was most unfortunate he had not been placed long ago, when his friends and neighbours first saw symptoms of insanity working within his mind. It was most unfortunate that the authorities did not take up the matter, and did not place a man under restraint who went about day after day talking about murdering his wife, not in the language of a sane man, but in a way which impressed every one who heard it with the fact that he was insane. If that had been the case, the dreadful event which they had heard described to them would never have happened. He (the learned counsel) therefore asked them to consider, after having surveyed all the circumstances of this case, whether they would not come to the same conclusion that all his neighbours had arrived at before the event—that the prisoner was not in his right mind. He would submit to them, that if a number of the prisoner's neighbours, who knew him in his days of health, before disease had operated on his mind—who knew him as a quiet, inoffensive, honest and industrious man, a fond husband and a good father, previous to the mania which had taken possession of him, and who knew that his mind had become suddenly perverted, and that his conduct was that of an insane man—he would appeal to them whether the circumstance of the man having not only threatened to kill, but had actually killed his wife, was not strong evidence of his insanity. It was impossible for him to exaggerate the importance of this inquiry. It was not a question simply of mercy—it was a question of justice. It was of the utmost importance to every one of them; for who knew whether, on the morrow, the finger of Providence might not be laid upon them—that disease might not touch their minds, and that they might not be reduced to the condition of that man? There was not one of them in the court who was not liable to such a visitation of Providence, and not one who might not be afflicted like that unfortunate man, and under that suffering and affliction which he endured, do precisely what he did. Therefore, seeing this, it was, he contended, of the utmost importance to him, to the jury, and to all of them, that they carefully discriminated between a criminal act, done with the intention of its being a crime, and an act done under the influence of a diseased mind, leading the man to the commission of an offence. He was quite aware of the prejudices—the wholesome prejudices—that existed against defences of insanity: and he would be the last man to set it up, unless he was satisfied that it was really and truly an honest defence. It was not, however, because sometimes this defence was improperly made use of, that therefore they should close their ears when the defence came properly before them, and substantiated by facts which would lead them to the conclusion—let it be total or partial insanity—that this man was not in the clear possession of his reason. He would now endeavour to explain, in familiar terms, what insanity was; and what it was under which this man laboured, and which the doctors called monomania. He hoped to explain, in an intelligible way, that as the mind of man became partially diseased, he became, so far as that disease was concerned, incapable of exercising the functions of a human being, who was responsible for his actions, and was, therefore, entitled to the lenient conclusions of the jury in consequence thereof. He believed that they would be enabled to apply this description to the case in question, and come to the conclusion that the prisoner was insane in reference to the point he had raised. He knew that there were many persons who believed that a man's mind could not be partially affected; but he believed he should be able to show them that it was so, and that those so affected could not see the results of actions in the same way as they saw them through the medium of an undiseased mind. What were they?

They had souls enclosed in bodies, and it was only by the arms, the eyes, the nerves, and the brain, that the soul held communication with the world without. Did they believe for a moment that that spark of divinity—that soul which God had given to them to participate in the inheritance of angels; that that soul became diseased, and that when a man was insane, the soul itself was insane?—that the soul of the idiot was a soul deprived of all its distinctive features, either of humanity or divinity? What was insanity? It was not that the mind was diseased, but only those organs were diseased through which the soul communicated with the world without. It depended entirely upon the healthy or diseased state of that medium of the brain through which the soul communicated with the world, what impressions were conveyed to it. He would give them a comparison which would enable them to understand his meaning. Suppose they were placed in a room where a window was made of different coloured glass. They looked at the landscape through a pane of clear, transparent glass; they then saw that landscape as it was—they saw the sky blue, the fields green, and the distant waters sparkling in the sunshine. They saw nature in all its beauty. Let them go to the next pane—let it be one of red. What did they see? It was the same eye that looked through it, and the same mind—but yet, how changed the landscape! The sky, which was before blue, was to them a vault of fire, the trees became flames, and the whole country assumed the aspect of a hell—and yet it was the same eye, looking through the same mind, at the same landscape, which was before, and was still, green and beautiful. It was the same with a man looking through a diseased organ—which distorted everything to his mind. It was true that all the other panes in the window of his soul were clear, and that the man could reason on most subjects as well as he or they could; but when the mind looked through the diseased organ, then all things became changed, truth became untruth, reality became unreality, and beauty became deformity,—he did not know right from wrong, nor fact from a dream. He was looking through a coloured glass, and the world without him was a hell wherever he looked. That was what was termed monomania, and he appealed to them, therefore, whether the facts and circumstances in this case did not lead them to the irresistible conclusion that the prisoner was a monomaniac; that the man saw through the coloured medium of a diseased brain, and that whenever that diseased brain was excited, he ceased to be himself, he ceased not only to be able to discern truth from falsehood, but to mistake dreams for realities; to believe that which he did not see, and to lose entirely the control of those passions which were influenced by that diseased organ. Let them look at the facts. Here was a quiet and inoffensive man, who was respected by all his neighbours and beloved by all his friends. Within four years all that suddenly changed, and that which was good and excellent before, became suddenly violent,—he dreamed dreams, saw visions, and believed them to be true. Here was a man jealous of his wife. He had no reason to be so, and if that were so, then it was a delusion, and he was mad on that subject. If there had been the slightest ground for the suspicion, would not the whole place have rung with it? He appealed to the jury that if, with their knowledge of human nature, criminal intercourse had taken place between Punchard and the deceased, whether Mrs. Punchard would not have been the first to have found it out, and whether she would have remained the friend of her husband's paramour? The single circumstance that Mrs. Punchard never suspected the deceased, and had continued her friend, was sufficient to satisfy them that the whole thing was a delusion. What then was a delusion? They did not talk of a healthy, undiseased man having a delusion? They must give to things a right name, and in doing so they must call this delusion madness. If they had seen friends labouring under monomania, they would understand it. If they had not seen this, probably they had seen a friend ill in a fever, who became delirious. What did he do? Why, was he not constantly harping upon one thing, perhaps for weeks, until a restoration of the diseased organs took place, and then it was that the delusion ceased. He asked them, therefore, to say that the prisoner was labouring under a delusion of the nature described, and to give him the benefit of it, and not doom him to the extreme penalty of death. The jury would recollect how the prisoner was teazed and taunted—how the wife had encouraged the delusion by her conduct towards her husband. This was a man not merely suspecting, but believing that he saw his wife committing the actual thing which he stated. He believed that he had seen it—that he had been on the house-top, and saw improper intercourse between his wife and Punchard. What more convincing proof could they have than that of the prisoner's imagining that he had seen this criminal intercourse? But the prisoner was charged with wilful murder, and that was defined as malice aforethought. But if that were so, did they ever hear of any man's committing it in the way the prisoner had? It was not done in the way that dreadful crimes were usually committed. It was proved that the prisoner had gone about saying he would do it, and was not that, he asked, a convincing proof of his madness? How did he do it? He went deliberately and murdered his wife, leaving the evidence of his guilt behind him. There was no attempt at concealment; for he afterwards went and told all about it to the brother of his wife. He believed that the jury would say that the prisoner was mad. Undoubtedly he was right in every other part of his mind, but he did not believe that they would send him to the gallows for an act, which resulted from the action of one part of his mind being clearly diseased. He further contended that if the prisoner was mad, then the offence was not murder, and if he actually saw what he stated that he did, and committed the dreadful act under the passions evoked by it, then it would not be murder but manslaughter. They would recollect that the man evinced no desire to fly from justice; he told them that he went and looked upon his dead wife, and felt sorrow for the crime. When the mania took place, he was excited, and ceased to have possession of his reason, or his faculties. Here it was on evidence that the mania had become abated, the excitement had gone off, and on looking at the foul work he had done, he 'was sorry.' The learned gentleman concluded by saying that he should prove that up to the time that this malady took possession of him, he was a quiet and inoffensive man; but whenever under the influence of this delusion, he raved, swore, and tore his hair, and was not in fact, a responsible being. The form of the verdict would be, if they believed him insane, 'not guilty on the ground of insanity.' By such a verdict, they would not let him loose on the world, when perhaps that mania might return again, and he might do other violent acts. The practical effect would be, to confine him during the Queen's pleasure for the remainder of his life. They would thus be doing no harm to society, but giving the prisoner a chance of being medically treated, and of being recovered from this diseased state of mind. On the other hand, if they believed that the prisoner committed the crime under the violence of uncontrollable passion, and with the belief that his wife was really guilty of that which he suspected, then they ought to find him guilty of manslaughter only. He adjured them to lean to the side of mercy, for the diseases of the brain were, unfortunately, of such a character that they were difficult to be decided upon. They could not open a man's skull and see whether the brain was softened or diseased. They ought, therefore, to incline to the side of safety, and consign the prisoner for the rest of his life, where he would be taken care of, and prevented from again going into society. He hoped, therefore, that under all the circumstances of this case, they would come to a verdict in favour of sparing the life of this unfortunate man. The learned counsel then called:—

*Rev. Isaac Henry Gosset*—I resided at Northam. I was incumbent, and knew the prisoner and his wife since 1844. I knew nothing wrong of them.

I left Northam in May last. I saw the prisoner in April, about an allotment he held from me, and I thought his manner strange and confused. He came with Philip Dennis, to make an arrangement about this allotment. Hancock wished to give up the allotment, and Dennis wished him not to do so, but that he might hold it for him for a year, to see if he would get over the delusion, in reference to Punchard, under which he was labouring. I was very much impressed on that occasion that he was out of his mind. He did not say anything about Punchard, but I knew to what he alluded when he said there was no use keeping it; so long as things remained as they were, he could not stick to anything. I was led to notice his manner, because I had formed an opinion about August last year that he was labouring under a delusion about Punchard. I was sent for to see Hancock, he was reported as ill, in July or August, 1854. On that occasion he was not at home, and I heard from his wife a long story about his delusion about Punchard. She said she considered herself in danger of her life. I directed, on that occasion, the constable to take him up, fearing that murder would be committed.

*Cross-examined*—Before May, when I left, I was continuously at Northam, since 1844. In 1854 I don't know if I had any conversation with the prisoner. I formed my impression that he was not of sound mind, from what I heard from his wife. In May, when I had the conversation with him about the allotment, I thought his manner changed. I heard, so far back as July and August previously, from more than one quarter, that he entertained suspicions against Punchard. In 1854, when I saw the wife, I believed the suspicions were groundless. The prisoner's manner, in April, 1855, was very confused. He did not appear to speak with ordinary intelligence.

Counsel—What was peculiar in his manner at that time you witnessed?

*Witness*—I cannot describe it; but it deeply impressed me. I have frequently heard of his ill-using his wife. At the time he was taken up at my suggestion, it was under an apprehension that murder might be committed. I thought he should be bound over to keep the peace, or sent to an asylum.

*Charles Edward Pratt*, physician at Appledore, said—I have charge of the Northam district of the Union, and knew the prisoner as long as he has been in the parish. I have been his professional attendant. In the earlier part of my acquaintance with him he was a quiet, civil, inoffensive man. I noticed a change in him about fourteen or sixteen months ago; I found him sullen. He used to pass me without speaking, and I remarked it to my son. I heard some complaints made of him, and I desired the parties to apply to Mr. Gould, the magistrate. Mr. Gould applied to me, and I went to see Hancock. I went on a Sunday morning, about Nov. 1854, about eleven or twelve in the morning. He was in bed, and unwilling to see me. He was low in spirits, and short in his answers. I ordered him some stimulant. Next morning he called upon me as I desired, my object being to ascertain the state of his mind. I brought up the subject of his wife's conduct, as I was aware that was a weak point with him; he told me what his eyes had seen he could believe, and mentioned some circumstances and occasions connected with the intercourse of his wife with Punchard. He said he had seen them in a donkey-house, and he could put his hand upon them, they were so close. I went and examined the donkey-house. He said he was in a pigsty at the time, and the tumble roof went into a donkey-house. When in the pigsty, he could not have reached to the donkey-house. The prisoner also said on another occasion that he had seen them in the same donkey-house from the roof of another house. On the roof he certainly could not have seen inside the donkey-house. Another time he said he had seen them in the passage at the back of his house. I made my report to Mr. Gould that he was labouring under a delusion from jealousy, but I saw no occasion to put him under restraint. His manner altogether was changed, but I could detect nothing wrong in his conversation.

By the Judge—I have no doubt in monomania; that a man may be perfectly sound except on one point.

*Examination continued*—After making my report to Mr. Gould, I thought I had taken too much responsibility upon myself, and I watched the case until he separated from his wife. My general impression as to the state of his mind was that he was mazed, that is not exactly mad, but tending to it.

By the Judge—Do you consider he could not distinguish between right and wrong?

*Witness*—I believe that when he was in a paroxysm he could not.

*Cross-examined*—I cannot say that ever I saw him in a paroxysm. The first time I saw him excited was when he came to my house. It was the day after I told his wife to give him some beer as a stimulant. When I called on the Sunday he was in bed, and I thought he was unwell. He had been suffering from diarrhœa, but I did not call on that account, but at the request of Mr. Gould to inquire into the state of his mind. When he came next day he entered as an ordinary person would. He was out of spirits—in the state a man would be in if something was preying on his mind. I had conversed with the wife before, and brought the subject of Punchard before the prisoner, to test the state of his mind. He stated to me quite distinctly where he had seen his wife and Punchard. The language he used implied that he had seen them in the act: but the precise words I cannot remember. He was with me on that occasion about fifteen or twenty minutes. He named places, and I afterwards proved their position, and his accuracy. I had noticed the change in his manner about the time the rumours originated. When I met him, and observed the change in his manner, it was about June or July last year, and that manner continued very generally down till this occurrence. I saw him several times afterwards, and generally inquired as to how he was getting on with his wife. I inquired among the neighbours also, and I found he had been threatening his wife; but believing he was quiet and inoffensive, I thought he might be safely left. I considered all the time that he had no cause for jealousy. I did not know at the time that his wife had said she preferred Punchard to him. On the same morning I saw the prisoner in bed, I saw Dennis, and said, "What do you say to Robert Hancock?" He said, "That is just what I was going to ask you." I said to him, "I can detect nothing, but I will look after him." I had not then discerned his delusion. After I had discerned the delusion, I did not say to Dennis, "He is no more mad than you, Philip, except that he is a jealous husband." I met Dennis frequently afterwards, but never, except on this occasion, had any conversation with him on that subject. I was at the inquest on the 3rd August. Was in the room the whole time. I gave no evidence on that occasion.

*Elizabeth Brown*—I live at Northam, aud have been neighbour to the prisoner for fourteen years; in the house adjoining for the last six or seven. He was a steady and quiet man till within the last two years. He then became jealous, and quarrelled with his wife about William Punchard. There was no cause for that jealousy. The first time he said anything to me about it, it was twelve months before August last year. His wife called me in, and in the prisoner's presence said she wished me to hear what Robert was upbraiding her with. "Robert," I said, "what do you mean?" He said—"My wife and Punchard is greater than they ought to be; she is a bad woman." I thought he was out of his mind, because the accusation he made I believed to be false. I saw nothing more to make me believe that he was not right in his mind.

By the Court—I said to the prisoner—"Why do you take this into your head?—it is false." He said he had seen them together. He said he had seen his wife with Punchard in Mr. William's linhay at five o'clock in the morning. This is between Appledore and Northam. He said also that he had seen them in the donkey-house, and in the dung-pit. I said—"Robert, that's impossible; because the water thrown in to make the dung, comes up close to the back door." He said he had seen them many a time. I spoke to Dr. Pratt frequently, because I was afraid he would commit murder. I did not think him in his right mind, but I did not perceive it on any other subject but that of jealousy of his wife.

*Cross-examined*—The prisoner and his wife left in February last, and I never was present at any quarrel after. I still often heard of quarrels. This jealousy arose between two and three years ago. He appeared to get more jealous latterly. In February, the wife and Hancock quarrelled and separated. I had seen a violent quarrel a few days before the 3rd of February, when he left. I have seen him the worse for liquor, but not often, and I think he was then more violent. I heard him say he had seen his wife and Punchard together on the 21st of January, but more than twelve months before that he told me that he had seen them together.

*Maria Mules*—I live at Northam, and know the prisoner. I have known him thirteen years. In April last I heard a noise in his house, and went in. He was standing between the fireplace and the stairs, and his wife beside him. He was like a wild man—like a mad person. I saw him take up a hatchet, and said he would destroy the wheelbarrow. He had been speaking about the wheelbarrow, and said that his wife had been down with it at Punchard's. I did not see the wheelbarrow chopped, but he went out, and I heard chopping afterwards. He asked me to go to bed with his wife, because he thought she was afraid to sleep in the house alone. He said he would sit up by the fire, and not go to bed, because Punchard would be in with his wife again. He was very violent, and asked his wife to give him poison. She declined to give him any. I don't know if any was in the house. He was pulling his hair very much, and pulled it out in handsful and threw it on the floor. He took off his handkerchief and unbuttoned his shirt, for the purpose of cutting his throat. He had no knife, but one was on the table, which I took up and kept till he had gone. He asked for a razor. His wife said she had concealed it; and she called to me to get assistance. I called for assistance, and Richard More came, who is not here, and Mr. Braund, the constable, who took him into custody. His wife said it was her wish he should go away, because Mr. Pratt said he was not right. I reckoned she meant he should go to the asylum. She said that when he came back she would live with him again. When I first knew the prisoner he was very quiet, and would not injure anybody.

*Cross-examined*—I did not know what had led to the quarrel that night. When I went in he had up the pocket of his wife's dress, and wanted to get her money. When I came in he left his wife, and went and sat in a chair, apparently in a great state of sorrow and grief. I don't know, but I think the grief was about Punchard. He said he would destroy the wheelbarrow, and the wife said—"There is the hatchet," which he took up and went out with. He sat down and smoked, and got better after I came in; but his wife was aggravating him very much, and said that Punchard was a better man than him.

*William Punchard*—I am a mason, and live at Northam. I knew the prisoner for seven years, and his wife also; but never had any unlawful intercourse with the deceased; I never was in the prisoner's house since he lived there. I never was with her on the dung-pit, in the pigsty, donkey-house, or any other place on the day of the murder. I was then confined to the house from an accident.

*Cross-examined*—I knew of this report of an intimacy between me and deceased, but I never heard the prisoner say anything about it in my presence. He told my wife, and she told me. He said in Mr. Gould's house that he was not going to hurt a hair of my head.

*Dr. Bucknill*, medical superintendent of the County Lunatic Asylum, said—I have heard the witnesses at this trial, and have had conversation with the prisoner. In personal conversation with the prisoner, which was only once, yesterday, I found him under a strong conviction that Punchard had committed adultery with his wife. His story, in support of that opinion, was altogether inconsistent with itself and almost incoherent. There were absurdities in it, and distinct and glaring inconsistencies. My opinion was that it was probable this was a delusion. He told me he had seen this adultery committed in several instances, and he expressed himself in very strong and excited language. He said it had occurred as often as he had hairs on his head,—thousands of times. He said he had actually seen it three times, but when questioned, he appeared to have seen it only once. He said that he had placed himself on the roof of a house and had actually seen it in the court at the back of his house, against the wall outside. He said, also, he had seen his wife meet Punchard and go with him into the passage, when they bolted the door. On the night of the murder, he looked out of his house, and saw Punchard come out of his door, and look up and down the street to see if any of them were about; and then seeing the coast clear, his wife slipped by Punchard. He then discovered himself, and said, "So here you are again." I ought to add that I was impressed by the strange state of feeling he evinced with respect to his present position. He repudiated the idea of being insane very strongly, and he said that when he was angry with his wife about her conduct, people said, "Oh! here you are with your old mazed tricks again." He spoke of the day of judgment; not of this judgment—this was a trifle—but of the day when he would meet Punchard, and he seemed to be under strong feelings of revenge, which would be gratified by Punchard's punishment. He said it was the last word he should say on the gallows, that Punchard was the ruin of his wife. Having heard the evidence, and assuming it to be true, my opinion is that he was labouring under monomania, but not under general insanity. Delusion is a fixed form of insanity, and monomania means that a person is entirely mad upon one point. On that subject he would be unable to distinguish between right and wrong. He might think in killing, when under such an influence, that he was doing a meritorious act. On the point of his delusion all the faculties, will, judgment, reasoning powers, &c., are affected. The prisoner seems to be under hallucination—to see and hear things which have no existence in fact.

*Cross-examined*—I first saw the prisoner yesterday evening, and was with him about an hour. I went to the jail with a very slight knowledge of the case. I was aware the proposed defence was insanity, and knew the point he was thought to be insane upon was with reference to Punchard. There remained on the prisoner's mind during the whole time a strong belief that Punchard and his wife had been guilty. A belief stronger than a sane man would entertain, except upon the most undoubted proof. A change may have taken place in the man's mind since August. His malady may have increased or decreased. His conversation with me was consistent with the supposition that he had seen what he stated, and it was consistent with the idea that it was all a delusion. But the story was inconsistent with itself.

Mr. Karslake, for the prosecution, summed up the evidence.

The Jury retired, and after being absent about an hour and a quarter, brought in a verdict of WILFUL MURDER, but acquitted the prisoner on the ground of insanity.

